# Simultaneous quasi-one-dimensional propagation and tuning of upconversion luminescence through waveguide effect

**DOI:** 10.1038/srep22433

**Published:** 2016-02-29

**Authors:** Dangli Gao, Dongping Tian, Xiangyu Zhang, Wei Gao

**Affiliations:** 1 College of Materials & Mineral Resources, Xi’an University of Architecture and Technology, Xi’an, Shaanxi 710055, China; 2 College of Science, Xi’an University of Architecture and Technology, Xi’an, Shaanxi 710055, China; 3 College of Science, Chang’an University, Xi’an, Shaanxi 710064, China; 4 College of Electronic Engineering, Xi’an University of Posts and Telecommunications, Xi’an, Shaanxi 710121, China

## Abstract

Luminescence-based waveguide is widely investigated as a promising alternative to conquer the difficulties of efficiently coupling light into a waveguide. But applications have been still limited due to employing blue or ultraviolet light as excitation source with the lower penetration depth leading to a weak guided light. Here, we show a quasi-one-dimensional propagation of luminescence and then resulting in a strong luminescence output from the top end of a single NaYF_4_:Yb^3+^/Er^3+^ microtube under near infrared light excitation. The mechanism of upconversion propagation, based on the optical waveguide effect accompanied with energy migration, is proposed. The efficiency of luminescence output is highly dependent on the concentration of dopant ions, excitation power, morphology, and crystallinity of tube as an indirect evidence of the existence of the optical actived waveguide effect. These findings provide the possibility for the construction of upconversion fiber laser.

Lanthanide ions based micro/nanomaterials have a wide range of applications such as displays^1^, *in vivo* and *in vitro* imaging^2^,^3^,^4^, the imaging of cancer cells and detection of biomarker molecules^5^,^6^,^7^,^8^, thermometer^9^ and enhancement for solar cell devices^10^,^11^. Despite these applications, it is necessary to solve the problem of remote propagation of the luminescence to further improve and expand their applications. However, the investigation on the remote propagation of upconversion luminescence is extremely lacking.

In recent years, more and more attention has been paid to the investigations of upconversion mechanism in micro/nanosystems^12^,^13^,^14^,^15^,^16^, tuning the laser parameters^17^,^18^,^19^,^20^,^21^, and designing new and more complex upconversion micro/nanostructures^22^,^23^,^24^,^25^ to obtain high upconversion efficiency and controlled spectral modulation. For example, core-shell structure successfully devideded space into activator doped region and sensitizer doped region to avoid harmful cross-relaxation^10^. While overheating effect could be weakened by separating emission region from absorption region on single micro/nanoparticle via a confocal local excitation^14^. In addition to, the local excitation of single paticles make some hidden physical effects clear. In 2011 years, Liu group designed a donor and acceptor spatially separated core-shell-shell structure^10^, where the excitation energy absorbed in the shell undergoes a long journey by energy migration between Gd^3+^ ions to reach the activators inside the core. The special energy migration process is belong to “long-range” interaction, and makes the distance of excited energy propagation be longer than 5 nm. However, the propagation distance is so small to some special application such as optical communication that it always is ignored.

It is well-known that, based on total reflection theory, waveguide generally is served as the optical signal propagation channel and optical interconnection at a long distance range^26^. Given the difficulties to efficiently couple light into a waveguide, various luminescence-based waveguides have emerged to directly produce the guided light under blue or ultraviolet light excitation^27^,^28^,^29^. Due to the unique one-dimensional (1D) structural characteristics and versatile physical/chemical properties, luminescence nanowires have emerged as the building blocks for a variety of fundamental optical components^30^,^31^,^32^,^33^,^34^,^35^,^36^,^37^,^38^. We also know that rare-earth (RE) doped NaYF_4_ compounds possessing a high refractive index and a low phonon energy have been regarded as currently one of the most excellent upconversion materials^2^,^12^. To my knowledge, someone tried to fabricate active waveguide in fluorite-glass composite by laser micro-machining^39^. However, 1D NaYF_4_ compounds as upconversion luminescent waveguides have not yet been reported. Compared to blue and ultraviolet excitations, near infrared light as excitation source has the higher penetration depth and less overheating effect and therefore is preferable for applications in optical communication and bioimaging^2^,^3^,^4^,^5^,^6^,^40^.

Here we show the design and realization of quasi-one-dimensional propagation of excited energy along the length direction of a single NaYF_4_:Yb^3+^/Er^3+^ microtube with a length of 55 μm. The propagration mechanism is attributed to the optical waveguide effect of the single microtube accompanied with energy migration. The effects of codopant concentration, excitation power, the size and crystallinity of microtube on the upconversion signal of individual microtube are also investigated in detail. The present investigation provides theoretical and experimental basis for designing special luminescent materials with targeted luminescence and optical waveguide effect, and has also brought a new idea in tuning multicolor upconversion emission.

## Results

### Structural characterization of NaYF_4_:Yb^3+^/Er^3+^ (20/2%) microtubes

**Figure 1:**
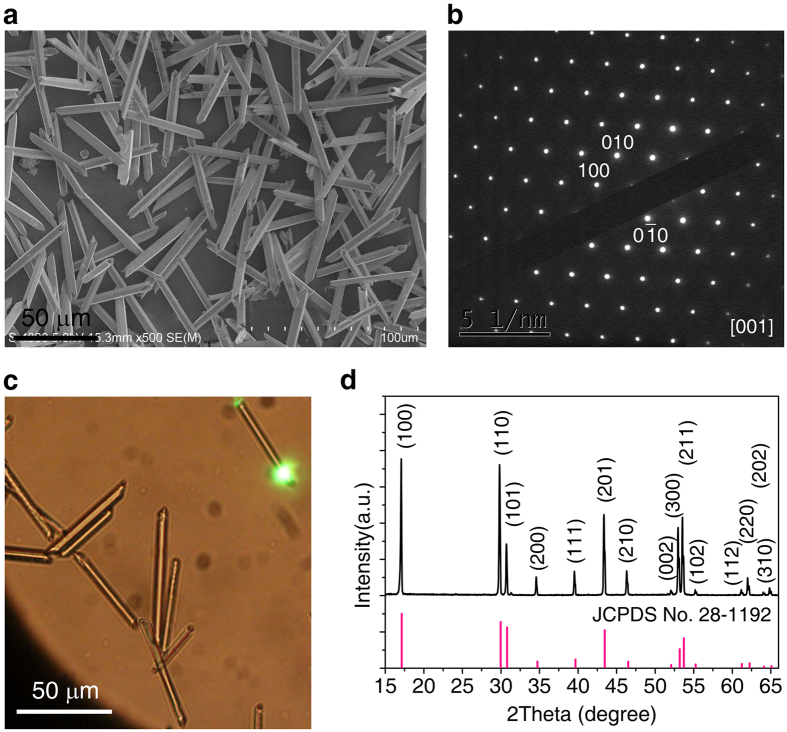
Structural characterization of the as-synthesized NaYF_4_:Yb^3+^/Er^3+^ (20/2%) microtubes. (**a**) Typical low-resolution SEM micrograph. (**b**) Selected-area electron diffraction pattern of a single microtube. (**c**) Bright field optical microscope image. (**d**) X-ray powder diffraction pattern of the as-prepared NaYF_4_: Yb^3+^/Er^3+^ microtubes showing that all peaks can be well indexed in accordance with hexagonal-phase NaYF_4_ structure (Joint Committee on Powder Diffraction Standards file number 28–1192).

NaYF_4_ microtubes codoped with Yb^3+^/Er^3+^ (20/2 mol%) were prepared by using hydrothermal method with the assistance of trisodium citrate^41^. The products were composed of uniform tubes with diameters of about 800 nm and lengths of about 5500 nm, as shown by the scanning electron microscopy (SEM) photo in Fig. 1a and bright field optical microscope image in Fig. 1c. The selected area electron diffraction pattern shown in Fig. 1b indicates the formation of NaYF_4_ monocrystal. X-ray powder diffraction pattern shows representative reflections for a hexagonal-phase NaYF_4_ with space group P63/m (Fig. 1d). Such highly crystalline tubes are resulted from the preferential growth along the [0001] direction (c-axis).

### Luminescence output from the top end and the centre of a single microtube

**Figure 2:**
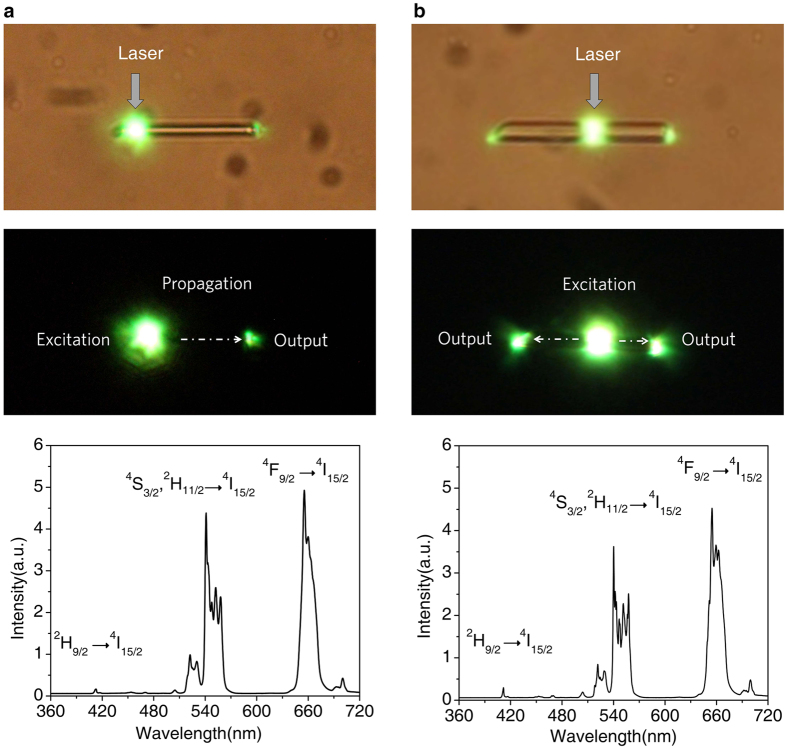
Bright field optical microscope images (top panels), real-color PL photographs (middle panels) and corresponding PL spectra (bottom panels) of a single NaYF_4_:Yb/Er (20/2 mol%) microtube with local excitation. (**a**) Local excitation at the central part of a single microtube. (**b**) Local excitation at one end position along its length of a single microtube. Scale bars for all images are 20 μm. All the samples were excited with a 980 nm focused laser beam operating at the power density of about 40 mW/cm^2^, in which the laser beam is perpendicular to the length direction of the tube.

Figure 2 top panels are the bright field optical microscope images of an isolated microtube on glass. The diameter and length of a single microtube were around 8 μm and 55 μm, respectively. Figure 2 middle panels are photoluminescence (PL) photographs showing the single tube being excited locally with a laser focused into a spot of about 1.0 μm in diameter at the end and the center of tube, respectively. The PL photographs indicate that the excited energy from the excited spot travels along the length direction of tube, which leads to the output of luminescence signals from the top end of a single microtube. Figure 2 bottom panels show PL spectra of a single NaYF_4_ microtube doped with Yb^3+^/Er^3+^ when the center and end of the microtube are locally excited by a focused laser beam of 980 nm, respectively. All the emission bands are assigned to Er^3+^ ions. Green luminescence corresponding to the ^2^H_11/2_/^4^S_3/2_ → ^4^I_15/2_ transition is located in the wavelength region of 500–575 nm, and the ^4^F_9/2_ → ^4^I_15/2_ transition derived from red luminescence ranges from 625 to 700 nm. These upconversion peaks in Fig. 2 bottom panels are in agreement with previous reports^42^,^43^,^44^. It is noted that the shapes and positions of peaks are similar when the single tube is excited at various positions by a focused laser beam.

Self-guided luminescence propagation to the distal tips can be clearly observed when different positions of the tube are excited by focused laser as shown in Fig. 2 top and middle panels. It is well-known that optical waveguide is a kind of device in which the high refractive index medium is surrounded by the low refractive index medium. This kind of novel local luminescence pattern of the single NaYF_4_ microtube, that is different from the previously reported luminescence pattern of the microtube where microtube is wholly illuminated^45^, indicates that NaYF_4_ microtube serving as a kind of new luminescence-based waveguide. Alternatively, we can image that complete reflection of a ray of light in a medium can only take place when the angle of incidence in microtube is greater than the critical angle of total reflection. As a result of total reflection, luminescence output from the tube end could be further promoted by improving the absorption of the excitation energy via increasing the path length of near infrared laser in microtube.

### The property of quasi-one-dimensional luminescence propagation

**Figure 3:**
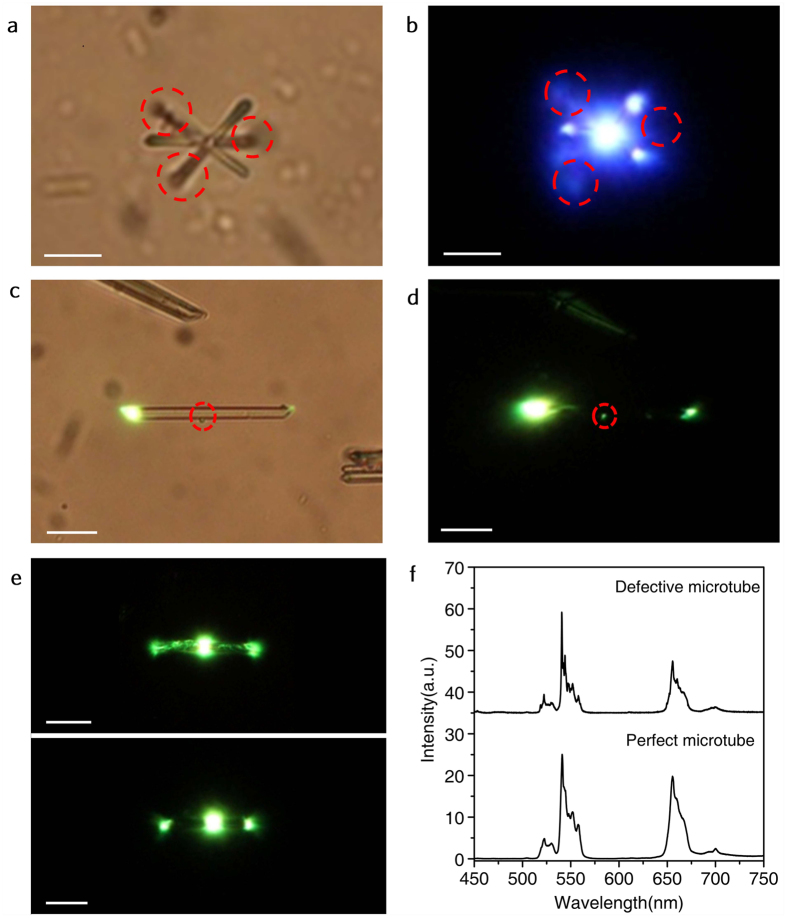
Bright field optical microscope images, real-color PL photographs and PL spectra of doped single one-dimensional upconverting NaYF_4_ microtube with 980 nm laser local excitation operating at 40 mW/cm^2^. (**a**) Bright field image from three crossed NaYF_4_:Yb^3+^/Tm^3+^ (20/0.5 mol%) microtubes. (**b**) Real-color PL photograph from three crossed NaYF_4_:Yb^3+^/Tm^3+^ microtubes by excitation at the point of intersection. (**c**,**d**) Bright field optical microscope images and real-color PL photograph from a single NaYF_4_:Yb^3+^/Er^3+^ (20/2 mol%) microtube obtained by excitation at one end of the tube. (**e**,**f**) PL images and their PL spectra from a defective and a perfect NaYF_4_:Yb^3+^/Er^3+^ microtubes by excitation at the central part. The dashed red circles indicate the defect positions of one-dimensional microtubes. Scale bars for all images are 20 μm.

Considering that the property of the luminescence propagation along the length direction is available for 1D NaYF_4_ rods with average length of 10 μm (Supplementary Fig. 1) that are synthesized through hydrothermal method in the presence of EDTA. Another added benefit is that the operating wavelength of upconversion waveguide in a wide spectral range can be readily adjusted by controlling the dopant composition. For example, blue and pink luminescence output from the top end of microrod can be easily obtained by codoping Yb^3+^/Tm^3+^ and Yb^3+^/Ho^3+^ ion pair, respectively (Supplementary Fig. 2). NaYF_4_ microtube/rod has special optical waveguide effect, which is important to extending their application such as upconversion fiber laser. As we expected that multiple microtubes of varying orientations as waveguide allows one to deliberately propagate light along bent lines as shown in Fig. 3a,b. A similar behavior was observed in CdSSe nanowire^29^. Noted that the luminescence leakage can be clearly observed in the propagation process due to the presence of the defects and the non-perfect geometries in Fig. 3a–e. So high-quality microtube/rod with smooth surface, uniform diameter and high crystallinity is necessary to the remote propagation of luminescence.

The reason for the quasi-one-dimensional propagation of excited energy could be complex in the single-tube under focused laser excitation. In a sensitizer-activator optical fiber system, the luminescence characteristics and energy propagation efficiency along the length direction of the tube is highly sensitive to the power density of the laser, the concentration of the sensitizer and the activator, and geometrical and physical parameters of tube.

### The tuning of red-to-green ratio (RGR) of luminescence output

**Figure 4:**
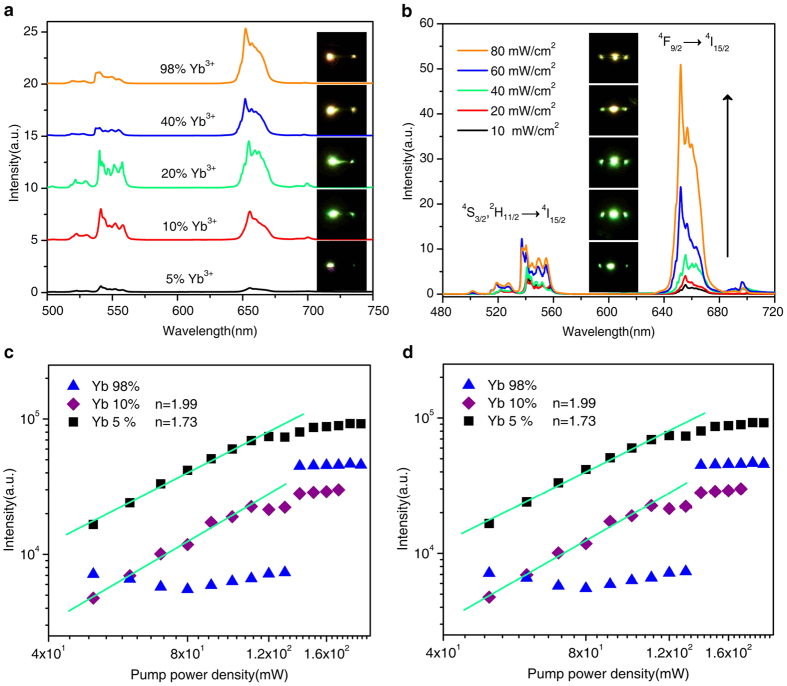
PL spectra and log-log plots of upconversion emission intensities as a function of excitation power densities from a single NaYF_4_:Yb^3+^/Er^3+^ microtube with focused laser excitation. (**a**) PL spectra of a single NaYF_4_:Yb^3+^/Er^3+^ (*x*/2 mol%, *x* = 5, 10, 20, 40, 98) microtube with a 980 nm focused laser excitation from the end part operating at 40 mW/cm^2^. (**b**) PL spectra of a single NaYF_4_:Yb^3+^/Er^3^ (20/2 mol%) microtube excited with a 980 nm laser from the central part at various power densities. (**c**) The log-log plots of green emission intensities as a function of excitation power densities from a single NaYF_4_:Yb^3+^/Er^3+^ (*x*/2 mol%, *x* = 5, 10 and 98) microtube with a 980 nm focused laser excitation at the end and center. (**d**) The log-log plots of red emission intensities as a function of excitation power densities from a single NaYF_4_:Yb^3+^/Er^3+^ microtube. The insets in (**a**,**b**) are real-color PL photographs of a single NaYF_4_:Yb^3+^/Er^3+^ microtube with a 980 nm focused laser excitation from the end part and the central part, respectively.

To further get insight of the properties of the quasi-one dimensional propagation of excited energy in a single-tube, we firstly measure the dependence of upconversion luminescence intensity on the concentration of Yb^3+^ ions and pumping laser power density when the laser is focused at local position of microtube. As shown in Fig. 4, the operating wavelength of the upconversion waveguide can be deliberately adjusted from green to yellow spectral region by controlling concentration of Yb^3+^ ions or power density. An increased RGR from 0.9 to 4.2 and from 0.7 to 2.2 can be obtained by elevating Yb^3+^ concentration and pump power, respectively (Supplementary Fig. 3). The tuning mechanisms by elevating Yb^3+^ concentrations can be easily attributed to the elevating of energy migration probability among Yb^3+^ and back-energy-transfer probability from Er^3+^ to Yb^3+ ^12,46,47.

RGR increases by elevating pump power, which is inconsistent with the previous reports in Yb^3+^/Er^3+^ doped NaYF_4_ nanocrystals, in which the RGR independent on power density under a diffusion laser excitation is assigned to a saturation effect of the energy transfer processes^48^. The contradiction may be related to excitation modes and excitation intensities. Theoretically, excitation density is directly related to the initial population of the excited states in an upconversion system, thence affecting the energy transfer process and the upconversion emission properties^12^. Yet there is no direct evidence to indicate that the energy transfer process depends on excitation power density as excitation density is relatively low. A relative low excitation power density is usually applied to the measurement of massive micro/nanoparticles under a diffusion laser radiation. However, for single micro/nanoparticle measurement under local excitation, high power density excitation is required since it can cause more Yb^3+^ ions in laser facula to be in the excited state until saturation in the micro/nanoparticles. Energy migration from interior to external of laser facula and a high level upconversion process, involving the population of green and red levels, has become an important approach of depopulation when the more Yb^3+^ ions are in the excited state. From this point of view, high excitation power density prefers energy migration and upconversion process, which is equivalent to the effect of higher doping levels of the sensitizer on energy migration, upconversion process and RGR. It is well-known that the length of energy migration is limited by diffusion constant and the lifetime and concentration of Yb^3+^ ions. As shown in Supplementary Fig. 2c,d, RGR tends to be stabilized due to saturation effect and the limted length of energy migration when further elevating pump laser power density up to 120 mW/cm^2^.

More interestingly, noted that the bigger size and better crystallinity of a single microtube also results in a higher RGB (Supplementary Fig. 4 and Fig. 3e,f), which is in contradiction to the previous result reported on codoped Y_2_O_3_^49^. Here the single tube acts as an active optical cavity and the luminescence will travel along the length direction of tube through incessantly repeated re-absorption and re-emitting processes^50^. During this active propagation, the re-absorption and re-emitting processes will continuously lead to the luminescence intensities ratio change by the cross-relaxation between ions^51^,^52^. The energy loss of luminescence mainly come from the non-radiative loss involved in each of the re-absorption and re-emitting process of Er^3+^ activator. While non-perfect geometries and defects lead to the luminescence leakage in propagation process along the length direction of tube, which reduce the path length of luminescence output and cross-relaxation between Er^3+^ ions. Therefore, the ratios of the luminescence output signals depend on microtube size, doping concentration and light path, which indirectly confirmed that a single NaYF_4_:Yb^3+^/Er^3+^ microtube belongs to active optic fiber. Note that the change of RGB by the scattering can not be fully excluded because we know the scattering of blue emission will be stronger compared with the red emission. While higher Er^3+^ ions concentration could lead to luminescence quenching in a laser facula and in propagation processes by cross-relaxation (Supplementary Fig. 5)^51^,^53^.

## Discussion

To well verify the mechanism of the population of ^4^S_3/2_ and ^4^F_9/2_ states under NIR irradiation, the upconversion luminescence intensity of green (^2^H_11/2_, ^4^S_3/2_ → ^4^I_15/2_) and red (^4^F_9/2_ → ^4^I_15/2_) emissions as a function of the pump power density was investigated. Figure 4 bottom panels show the log-log plots of the emission intensities as a function of excitation power density for the green and the red emissions, respectively. It can be seen that as the pump power density was lower than 100 mW/cm^2^, the emission intensity of upconverson increased with power obeying a rule of 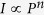 for both the green and the red emissions, where *I* is the emission intensity, *P* is the excitation laser power density, and *n* is the number of photons^54^,^55^. In addition to, the slope in the log-log curves varied with the Yb^3+^ concentration. Apparently, the value of *n* firstly increased and then decreased with increasing the Yb^3+^ concentration in the range of 5–98%. It can be also seen that as the power surpassed certain threshold, depending on Yb^3+^ concentration, UCL tend to stabilize. In other words, as the excitation power increases to a certain value such as 120mW/cm^2^, the power dependencies do not follow simple power law (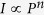). The upconversion luminescence intensity deviates from a linear relationship and the values of *n* decrease with power increase, which can be explained well by the “saturation” effect^54^,^55^,^56^.

The reasons for the *n* value modified by varying Yb^3+^ concentrations could be complex. The complications in the current study could stem from two sources: one is that energy migration channels are introduced as elevating Yb^3+^ concentrations, the other is that the three-photon upconversion process could be involved for populating the red and green luminescence levels.

In present case, when laser was focused at the position of the center or the end of tube, the Yb^3+^ ions that are within the laser facula would absorb photon energy of pumping laser and transfer energy to Er^3+^ ions and Yb^3+^ ions outside the laser facula. The excited energy density wasted by energy migration between Yb^3+^ ions would reduce the slope of *I-P* log-log curve, while high-level upconversion processes increase the slope. The smaller the laser facula and the higher the concentration of Yb^3+^ ions is, the larger the energy density wastes caused by energy migration. If the laser facula was larger than the energy migration length, the energy density waste caused by energy migration would be ignored. In our case, the slope of log-log variation *I-P* curves of two-photon upconversion firstly increase and subsequently violently decrease with Yb^3+^ concentration elevating, which can be mainly attributed to the competition between three-photon upconversion process and energy migration process for wasting the excited energy density within the laser facula.

Generally, the pumping laser power density could be written as 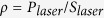, when pumping laser power is *P*_*laser*_ and its facula cross-area is *S*_*laser*_. Thus, the luminescence intensity *I* of two-photon luminescence is about 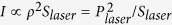
57. If this upconversion mechanism of NaYF_4_:Yb^3+^/Er^3+^ excited by a 980-nm laser is “energy migration among Yb^3+^ ions and then followed by energy transfer between Yb^3+^ and Er^3+^ ions sequentially”, the upconversion occurs not only within the laser facula but also in the range of luminescence facula determined by the diffusion length. In such a case, the intensity of two-photon luminescence is about 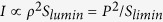. The ***S***_*lumin*_ denotes luminescence facula cross-area at the vicinage of excited spot. In such case, 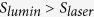 leads to a significant reduction of the real laser power density. In the log-log plot of the emission intensity as a function of excitation power density, if power density 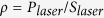 is much bigger than the real laser power density 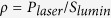, a significant reduction of the *n* value can be observed.

The higher excitation density and concentration of Yb^3+^ ions cause more Yb^3+^ ions to be in the excited state within the range of the length of energy migration, and the critical step in upconversion emission is the excited state energy transfer from Yb^3+^ to the activator Er^3+^. If the number of Er^3+^ ions is not enough, these Er^3+^ ions will get saturated easily in accepting excitation energy via Yb^3+^ ions. From this point of view, the higher the concentration of Yb^3+^ ions is, the lower the power threshold of saturation effect is. Therefore, when the concentration of Yb^3+^ elevating to 98%, even if under 50 mW/cm^2^ power density excitation, saturation effect could be observed. However, before reaching saturation of the Er^3+^ ions, more excited state Yb^3+^ ions are conducive to three-photon upconversion process of Er^3+^ ions when elevating Yb^3+^ concentrations from 5% to 20%, which contaminates the red and green luminescence level resulting in an increase of *n* value. As a result of the competition between three-photon upconversion process and energy migration process, *n* values firstly increase and then decrease with elevating the Yb^3+^ concentration.

In addition to, color center may also be formed at the femtosecond focused laser focus in microtube, which may further reduce the value of *n*. In fact, we found that the optical output signals from its both ends can be enhanced by nearly two orders of magnitude, when the microtube is locally radiated with 980 nm focused laser with a 120 mW/cm^2^ for 10 minutes in turn at various positions along its length (Supplementary Fig. 6 for the corresponding PL spectra). It is still not clear whether the luminescence enhancement is related to color center formed by laser radiation.

**Figure 5:**
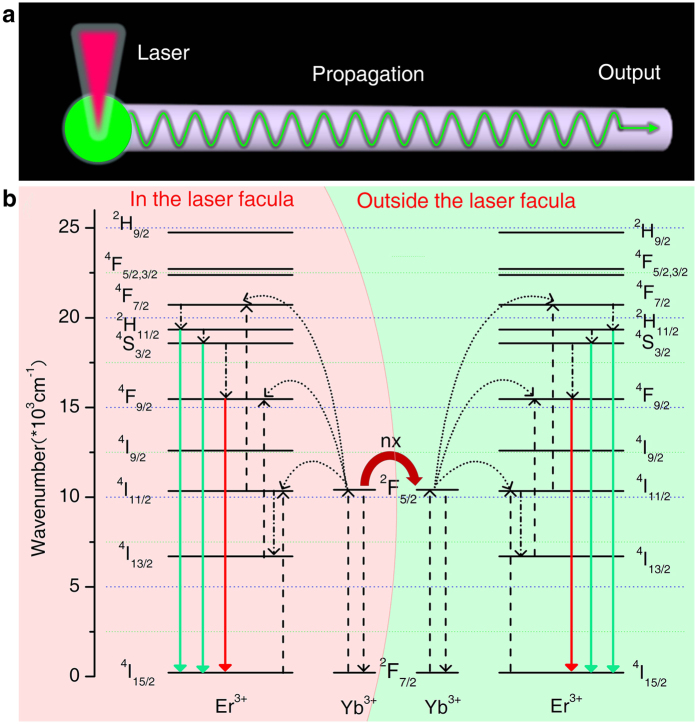
Schematic diagrams of a single NaYF_4_:Yb^3+^/Er^3+^ microtube as actived optical waveguide and the corresponding upconversion mechanisms of self-guided luminescence. (**a**) Schematic illustration of self-guided luminescence propagation, in which green circular region stands for upconversion luminescence facula rather than focused laser facula. (**b**) Proposed mechanism of self-guided luminescence production. Note that only partial energy levels of Yb^3+^ and Er^3+^ are shown for clarity.

Figure 5 displays the schematic illustration of light propagation mechanism and the corresponding upconversion mechanism for a single NaYF_4_:Yb^3+^/Er^3+^ microtube. Also indicated is the most relevant transitions that occur: energy migrations and transfers, cross-relaxation, as well as radiative and nonradiative processes. The upconversion processes are as follows: first, the Yb^3+^ ions within the laser facula absorb pumping photon energy and are excited to ^2^F_5/2_ state from ^2^F_7/2_ ground state. Then, the Yb^3+^ ions transfer energy to Er^3+^ or another Yb^3+^ ion outside the laser facula. The reciprocal action and energy migration among Yb^3+^ ions is rather strong with the high Yb^3+^ concentration. Not only two adjacent Yb^3+^ ions combine tightly to form a coupling state of cluster, but also there exists some long-distance correlation in the range of the diffusion length and laser elastic scattering region, which leads to bigger luminescence emission area than laser area with about 50 nm in diameter. The luminescence from in/outside the laser spot is propagated along the length direction of the tube due to waveguide effects of microtube, which leads to a strong luminescence output from the distal tips of a single microtube (Fig. 5a). That is to say, the characteristic saturation phenomenon of upconversion luminescence found in this study is mainly due to energy migration under local excitation. The typical saturation caused by population exhaustion has only a quite subordinate action.

Significantly, in this work, we have showed the design and realization of splendid quasi-one-dimensional upconversion luminescence output from the top end and the centre of Yb^3+^/Er^3+^ doped single NaYF_4_ microtube spin-coated on a glass slide under confocal optical microscope system. This specially designed one-dimensional structure allows the separation of excitation and emission regions of a single microtube. By monitoring the emission of a single microtube that is outside the laser facula, energy migration processes could be readily followed. The mechanism of the quasi-one-dimensional luminescence propagation is explored and is attributed to the optical waveguide effect associated with total internal reflection of the upconverted light at a single microtube in the medium-air interface. The upconversion output signal of an individual microtube is highly dependent on the concentration of dopant ions, excitation power, morphology, and crystallinity of tube.

Interestingly, we have found that the bigger size and better crystallinity of a microtube prefers to the higher ratio of red to green luminescence intensity in a single microtube, which is attributed to the reduce of the luminescence leakage. NaYF_4_:Yb^3^Er^3+^ microtubes as active optical waveguides not only violently absorb the incident excited light without the need for special alignment of the near infrared irradiation, but also propagate luminescence along the length direction of tube. The new physical effect of a single NaYF_4_ microtube, separated emission from excitation space, produces super bright local luminescence output at the top end and the centre of tube, which may be extending their application in the fields of upconversion waveguide laser, optical communication and biology.

## Methods

### Synthesis of microtubes

The NaYF_4_:Yb^3+^Er^3+^(Tm^3+^) microtubes have been fabricated via a facile hydrothermal route assisted by trisodium citrate^41^. In a typical procedure, 1.5 mL trisodium citrate (0.4 mol L^−1^) was added to a mixture containing 0.6 mmol RE nitrate (3.0 mL of 0.2 mol L^−1^ RE(NO_3_)_3_, RE = Y, Yb, Er, and Tm) and 20 mL deionized water. The solution was then thoroughly stirred for 30 min to form a chelated RE citrate complex (1:1 molar ratio for Cit^3−^-RE^3+^). Then, 6 mL (1.0 mol L^−1^) NH_4_F aqueous solutions were dropped into chelated RE Cit^3+^ complex under thorough stirring. The pH value of the mixture was tuned to 8.5 with ammonia water. Subsequently, the milky colloidal solution was transferred to a 40.0 mL Teflon-lined autoclave, and heated at 200 ^°^C for 24 h. The final products were collected by centrifuging and washed with water and ethanol. The collected microcrystals were dried under 70 ^°^C for 12 h.

### Synthesis of microrods

The NaYF_4_:Yb^3+^/Er^3+^ (Tm^3+^or Ho^3+^) microrods have been fabricated through hydrothermal method in the presence of the chelating agent EDTA^42^. Briefly, 0.75 mmol of EDTA (0.5 mol/L) was added to a mixture containing 0.3 mmol of RE nitrate (1.5 mL of 0.2 mol/L RE(NO_3_)_3_, RE = Y, Yb, Er, Ho and Tm) and 15 mL deionized water. The solution was then thoroughly stirred for 30 min to form a chelated RE-EDTA complex. Then, a solution 8 mL (1.0 mol/L) of NH_4_F and 4 mL (1.0 mol/L) of NaF aqueous solutions were dropped into chelated RE-EDTA complex under thoroughly stirring. Subsequently, the milky colloidal solution was transferred to a 40.0 mL Teflon-lined autoclave, and heated at 200 ^°^C for 24 h. The as-prepared products were collected by centrifuging and washed with water and ethanol for several times. The collected microcrystals were dried under 70 ^°^C for 12 h.

### Structural characterization

The phase compositions of the as-prepared products were examined by XRD with a D/Max2550VB+/PC X-ray diffraction meter with Cu Ka (40 kV, 40 mA) irradiation (λ = 0.15406 nm). The SEM micrographs were obtained using a Quanta 200 SEM. The selected-area electron diffraction measurements were performed using a JEM 2100 transmission electron microscope (TEM) operating at an acceleration voltage of 200 kV.

### Optical characterization

A confocal optical microscope system was employed for microscopic studies of β-NaYF_4_:Yb^3+^/Er^3+^ microtube spin-coated on a glass slide. The schematic diagram of the confocal microscope system is shown in Supplementary Fig. 7. Ti sapphire femtosecond laser (Mira-900) is employed as excitation sources, which passed through a half-wave-plate and a high reflective mirror at 980 nm, and then was focused with a high numerical aperture microscope objective lens (NA = 1.40, 1000×, oil immersion) to a spot diameter of about 50 nm. Emission spectra were collected and sent to the detection part after spatial and spectral filtering, and then detected by a spectrometer (SP2750i) with a spectral resolution of 0.008 nm equipped with a liquid nitrogen cooled CCD detector. All of the spectroscopic measurements are carried out at room temperature.

## Additional Information

**How to cite this article**: Gao, D. *et al*. Simultaneous quasi-one-dimensional propagation and tuning of upconversion luminescence through waveguide effect. *Sci. Rep.* **6**, 22433; doi: 10.1038/srep22433 (2016).

## Supplementary Material

Supplementary Information
