# Supplementary material for: Simultaneous quasi-one-dimensional propagation and tuning of upconversion luminescence through waveguide effect
Source: Sci Rep. 2016 Feb 29;6:22433. doi: 10.1038/srep22433 (PMC4772630; doi:10.1038/srep22433)
Supplement: Supplementary Information [file srep22433-s1.pdf]

Supplementary information

Simultaneous quasi-one-dimensional propagation and tuning of upconversion luminescence through waveguide effect

Dangli Gao[1,2,*], Dongping Tian[1,2], Xiangyu Zhang[3], Wei Gao[4]

[1]College of Materials & Mineral Resources, Xi'an University of Architecture and Technology, Xi'an, Shaanxi 710055, China.

[2]College of Science, Xi'an University of Architecture and Technology, Xi'an, Shaanxi 710055, China.

[3]College of Science, Chang'an University, Xi'an, Shaanxi 710064, China.

[4]College of Electronic Engineering, Xi'an University of Posts and Telecommunications, Xi'an, Shaanxi 710121, China. Correspondence and requests for materials should be addressed to D.G. (email: gaodangli@163.com).

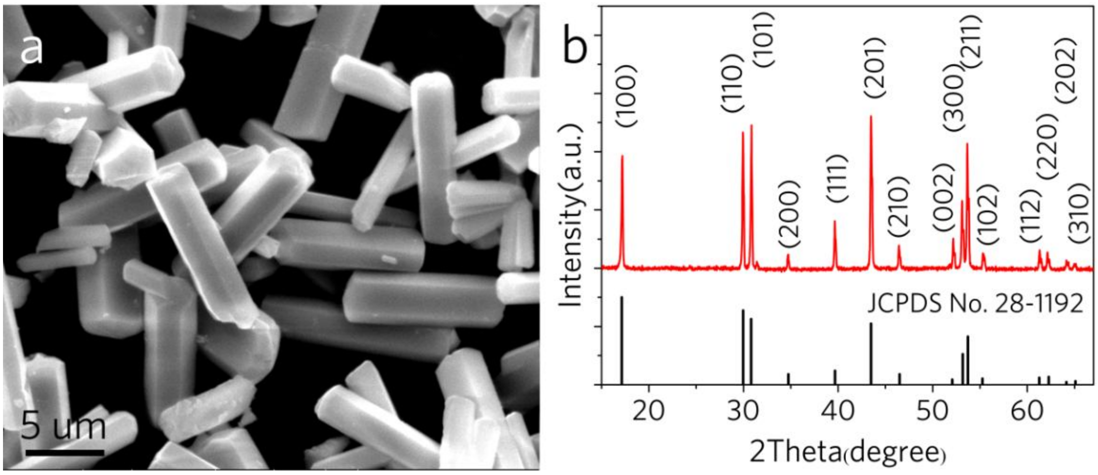

Figure S1. (a) SEM image of the as-synthesized $NaYF_4$: Yb/Er (20/1 mol%) microrods. (b) XRD pattern of the microrods and literature data for hexagonal phase $NaYF_4$ (JCPDS file number 28-1192).

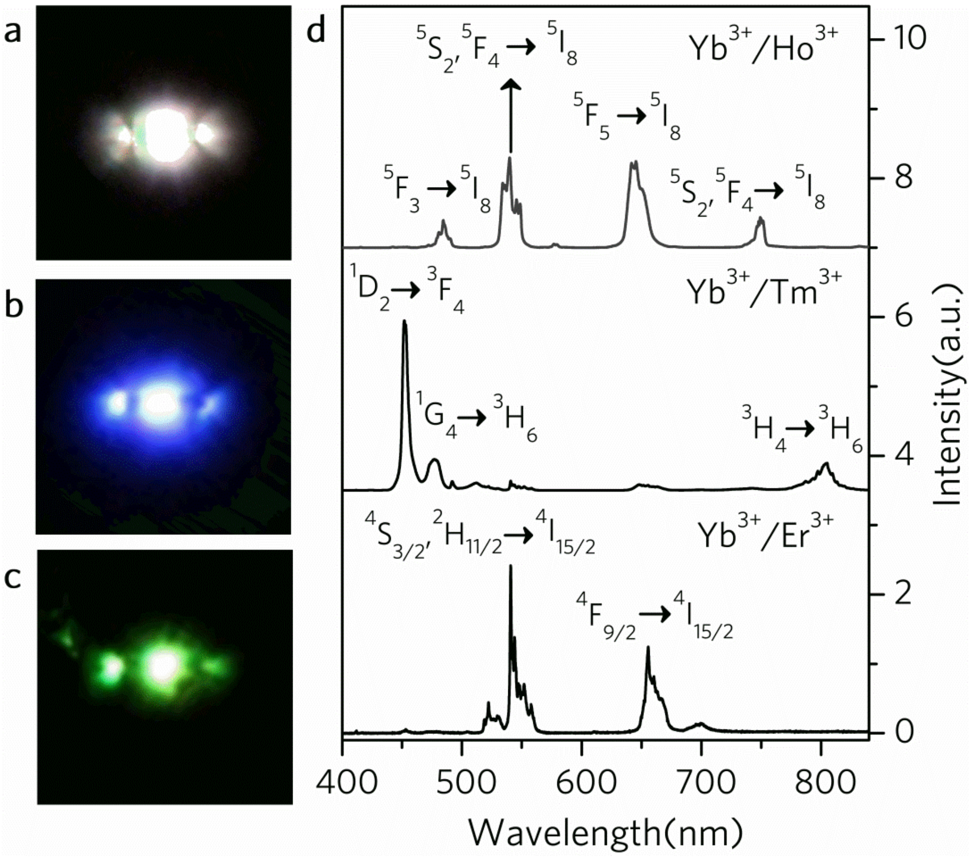

Figure S2. Real-color PL photographs and corresponding PL spectra of doped single $NaYF_4$ microrod excited by focused laser at the center of the rod. (a) Real-color PL photographs of single $NaYF_4:Yb^{3+}/Ho^{3+}$ (20/1 mol%) microrod. (b) Real-color PL photographs of single $NaYF_4:Yb^{3+}/Tm^{3+}$ (20/0.5 mol%) microrod. (c) Real-color PL photographs of single $NaYF_4:Yb^{3+}/Er^{3+}$ (20/1 mol%) microrod. (d) PL spectra of these doped single $NaYF_4$ microrod excited by focused laser at the center of the rod. All the samples were excited with a 980 nm focused laser operating at 40 mW/cm^2.

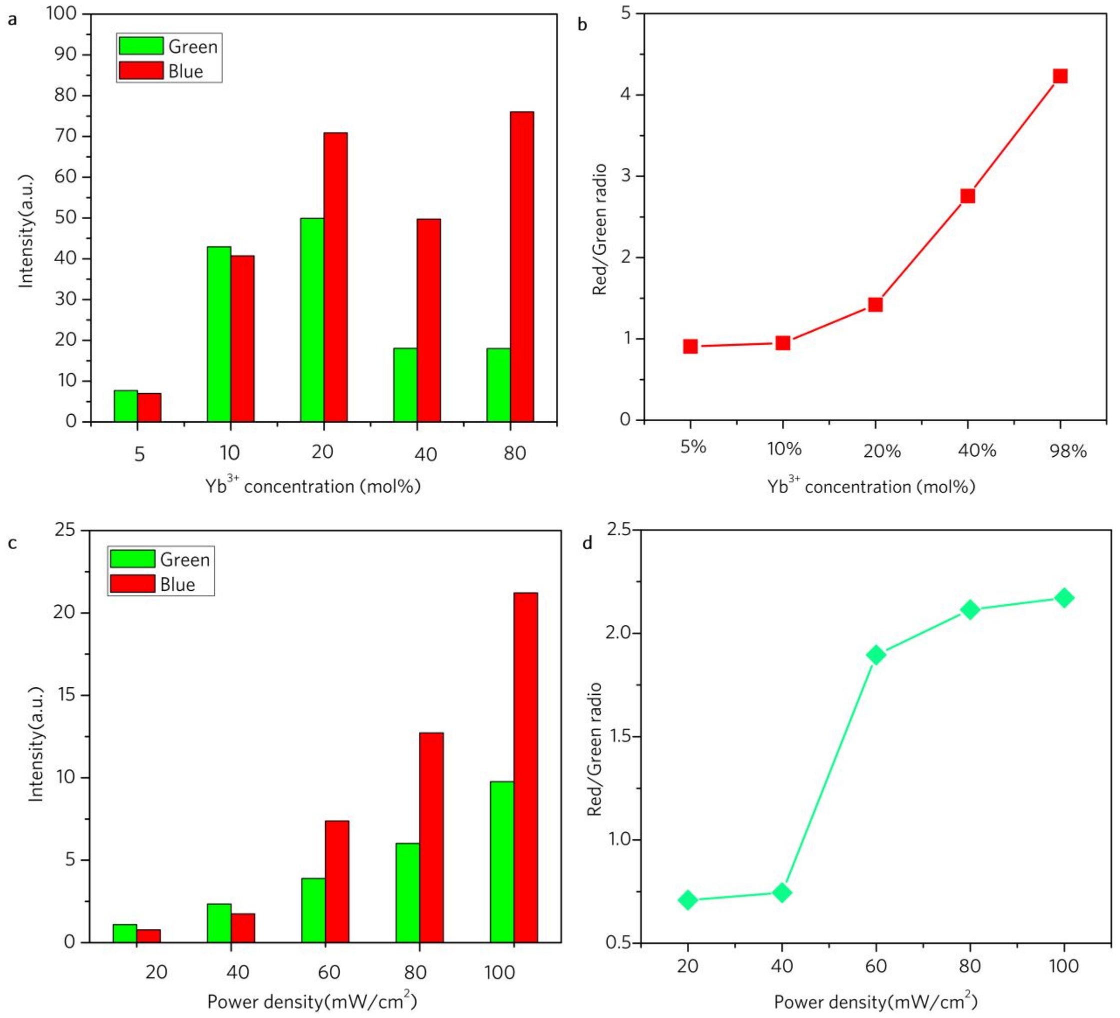

Figure S3. (a) The integrated intensity of the green and red emission of single $NaYF_4:Yb^{3+}/Er^{3+}$ (20/2 mol%) microtube as a function of Yb^{3+} concentration. (b) The responding RGR ratio of single $NaYF_4:Yb^{3+}/Er^{3+}$ (20/2 mol%) microtube as a function of Yb^{3+} concentration. (c) The integrated intensity of the green and red emission of single $NaYF_4:Yb^{3+}/Er^{3+}$ (20/2 mol%) microtube as a function of power density. (d) The responding RGR ratio of single $NaYF_4:Yb^{3+}/Er^{3+}$ (20/2 mol%) microtube as a function of power density.

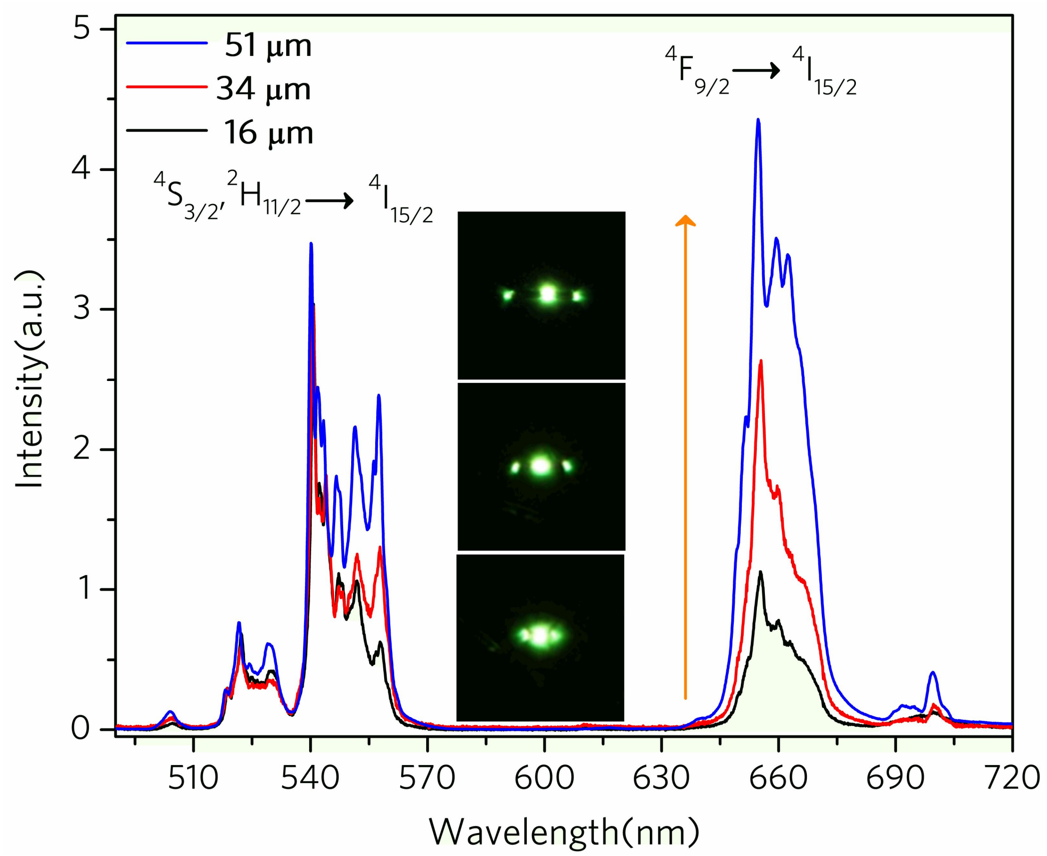

Figure S4. Real-color PL photographs (left panel) and corresponding PL spectra (right panel) of single $NaYF_4$ microrods excited by focused laser operating at 20 mW/cm^2 at the center of the $NaYF_4$: Yb/Er (20/1 mol%) microtube. The bigger particle has a much higher emission intensity in the red.

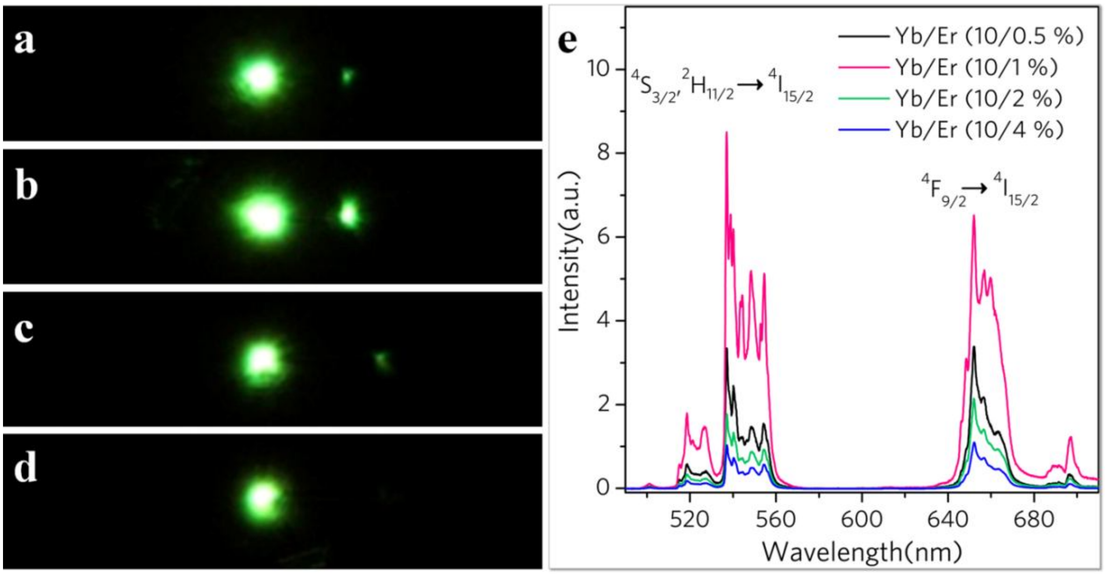

Figure S5. Real-color PL photographs (left panel) and corresponding PL spectra (right panel) of single $NaYF_4$:Yb^{3+}/Er^{3+} (10/x=0.5, 1, 2 and 4 mol%) microtube with focused laser excitation at the rod ends, operating at 20 mW/cm^2.

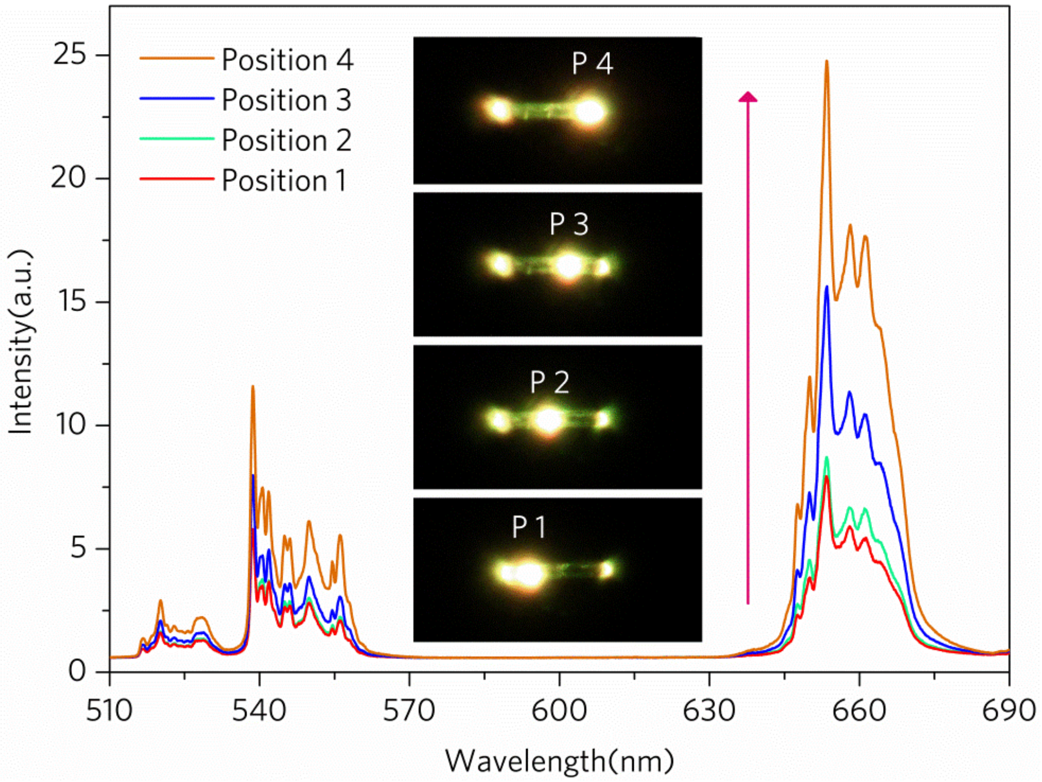

Figure S6. PL spectra of single $NaYF_4$:Yb^{3+}/Er^{3+} (40/2 mol%) microtube microrods, with local excitation at different spots (P1–P5) along its length. The inset display the corresponding real-color PL photographs. All the spots were excited with a 980 nm laser operating at 100 mW/cm^2.

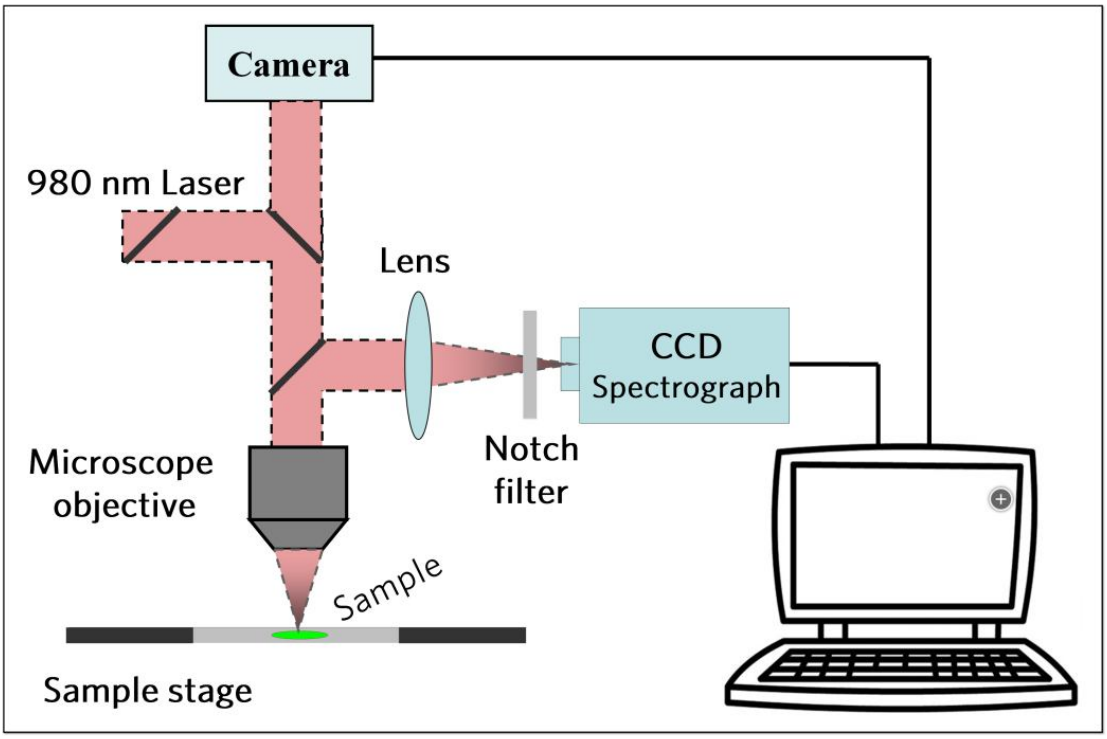

Figure S7. Schematic diagram of the luminescence spectroscopy test system.
